# BART Online Open-Source Sequence Toolbox for Computational MRI

**Published:** 2026-09-29

**Authors:** Daniel Mackner, Philip Schaten, Markus Huemer, Viktoria Buchegger, Moritz Blumenthal, Xiaoqing Wang, Martin Uecker

**Affiliations:** 1 Institute of Biomedical Imaging, Graz University of Technology, Graz, Austria; 2 Athinoula A. Martinos Center for Biomedical Imaging, Department of Radiology, Massachusetts General Hospital, Charlestown, USA; 3 Department of Radiology, Harvard Medical School, Boston, Massachusetts, USA; 4 German Centre for Cardiovascular Research (DZHK), Partner Site Göttingen, Göttingen, Germany; 5 BioTechMed-Graz, Graz, Austria

**Keywords:** Open-source, sequence development, model-based reconstruction, reproducibility, quantitative MRI

## Abstract

**Purpose:**

In advanced computational MRI techniques, acquisition and reconstruction techniques are jointly designed. For reproducibility, it is therefore important to provide an open implementation of both. At the same time, any use in a clinical environment usually requires a close integration with the MRI scanner. Ensuring long-time reproducibility and maintenance then poses additional challenges. In this work, we aim to provide a fully integrated open-source framework that can meet these demands.

**Methods:**

A software framework to develop pulse sequences is added to BART , an open-source toolbox for computational MRI. In addition, a vendor-specific driver sequence is developed that can be used to run the sequence on a clinical MRI scanner enabling online adjustment of all relevant sequence parameters. Using the Pulseq format, the exact same sequence can also be reproduced offline using a widely used vendor-neutral open-source standard. As proof-of-concept, quantitative MRI methods for *T*_1_ and joint water/fat $R_{2}^{*}$, *B*_0_ mapping using radial FLASH and model-based reconstruction are implemented in the proposed framework. Consistency between online and offline acquisition is validated in phantom and in vivo experiments.

**Results:**

Quantitative MRI methods highlighting specific challenges of acquisition and reconstruction were successfully implemented in BART. Acquisition parameters and FOV can be adapted online on a clinical MRI system. Quantitative parameter maps from model-based reconstruction agree for online and offline regenerated Pulseq acquisitions.

**Conclusion:**

This work enables reproducibility of advanced computational MRI methods within a comprehensive end-to-end open-source framework.

## 1 Introduction

Over the last decades, many new MRI techniques were developed to achieve faster acquisitions, higher resolutions, and to enable entirely new applications, especially for quantitative MRI (qMRI). With increased complexity of both, pulse sequences and reconstruction algorithms, reproducibility of published methods requires substantial effort due to missing details. For this reason, reproducibility became an important topic within the MRI community [1–4].

Pulse sequences are typically implemented using a vendor-specific proprietary platform, hampering translation across systems and sharing of source code within the research community. To overcome these limitations, several standards and frameworks for sequence development have been developed [5–7]. While many frameworks did not find traction in the community so far, Pulseq [8] together with pypulseq [9, 10], became a vendor-neutral open-source standard for sharing MRI sequences. Numerous applications have been published using the format, including MR fingerprinting [11, 12] cardiac *T*_1_ mapping [13, 14], and Chemical Exchange Saturation Transfer (CEST) [15]. Pulseq files are human-readable .seq-files that can be executed on MRI scanners of different vendors using vendor-specific driver sequences and can be used as input for Bloch simulations [16–18]. Environments for open-source pulse sequence development now usually offer the possibility to convert to Pulseq [19, 20]. While highly flexible in the sequence design and fully reproducible, the Pulseq format represents a pre-computed MRI sequence with limited possibilities to adapt the scan to each patient, which hampers its use in clinical studies. gammaSTAR [21] is a vendor-agnostic framework for pulse sequences that allows online adjustment of sequence parameters, but it is not open source.

In this work, we describe an open-source sequence programming framework, which is developed as an extension of the Berkeley Advanced Reconstruction Toolbox (BART) [22]. BART is an open-source toolbox for computational MRI. It includes algorithms for iterative optimization, efficient calibration and image reconstruction for parallel imaging and compressed sensing, advanced model-based reconstruction for quantitative MRI, as well as for machine learning. In addition to this basic framework, we developed a driver sequence that allows online configuration of the sequence on the MRI scanner. Sequences can also be exported to Pulseq files and later reproduced exactly.

To validate feasibility of the framework for development of advanced computational MRI techniques, we implemented two quantitative methods that combine radial acquisition with model-based reconstruction [22–30]. In these methods, the model-based reconstruction is sensitive to the specific details of the acquisition such as k-space trajectory and timing (*T_I_, T_R_, T_E_*), highlighting the need for an integrated approach. Reproducibility of these techniques is validated in phantom and in vivo experiments by comparing the results when using the online driver sequence with results obtained when reproducing the acquisition using exported Pulseq files.

## 2 Architecture and Implementation

A schematic overview of the BART sequence framework can be found in Figure 1.

### 2.1 Sequence Definitions

The specific configuration of an MRI sequence depends on a high-level parameter set, comprising fixed system parameters as well as adjustable parameters. System parameters include the maximum gradient strength, slew rate, or raster times. Adjustable parameters include geometric information (FOV, base resolution), the typical timing and contrast-related settings (*T_E_/T_R_*, flip angle), magnetic preparations (inversion pulse, *T_I_*, delays), as well as high-level dimensions (spokes, slices) and phase encoding settings. Similar to other sequence development frameworks, we compose MRI sequences from different event types, namely gradient events, RF pulses, sampling (Analog-to-digital converter, ADC) periods, wait and trigger events, which we each parametrize by start-, mid- and endtime, and additional type specific parameters.

**Gradients** are decomposed into triangles, each described by its amplitude at midtime in phase-, read- and slice-encoding direction. Hence, typical trapezoidal gradients have a compact representation using two overlapping asymmetrical triangles, while arbitrary gradient shapes can be designed by superposition of multiple gradient events corresponding to a linear spline interpolation. The MRI scanner requires that the gradient is computed on a discrete raster which is done by integrating the linear splines over each interval to obtain the average gradient for each interval. Using this average as gradient amplitude for each sample ensures that the zeroth moment is preserved in the descretized gradient wave form.

**RF events** describe a pulse with flip angle, phase, a frequency shift, and a mapping to a pulse shape. Pulse shapes are prepared prior to sequence execution. This preparation includes SAR/PNS safety checks (with duration, shape and number of pulse applications) where parameters can be adapted using the vendor-specific solve handler if limits are exceeded. In addition, we perform feasibility checks for the applied RF amplitudes, where we ensure that the amplitude is not reduced due to hardware limitations. A frequency offset and phase can be configured for the event, where the phase is defined for the midtime, with respect to the symmetry of the pulse shape. Nonlinear phases (e.g. for adiabatic pulses) are described with complex-valued RF samples. Pulse events are prepared on demand based on a mapping to previously prepared shapes.

**ADC events** are defined with a dwell-time and number of samples, as well as frequency and phase information, again using the midtime as a reference for the phase. ADC samples are located at the center of the dwell time intervals. In addition, each ADC event includes a vector of loop indices which are then stored together with the acquired data.

**Trigger/wait events** can be used to synchronize the measurement with external devices, i.e. by waiting for a specific condition or by sending a signal to an external device. Wait events can be used to insert delays. Parameters for those events are extracted from the respective timing information.

A set of events is combined into a sequence block, which are classified into magnetic preparation or imaging blocks. All events in a block are prepared as a function of the sequence parameters and the current sequence state. This state depends on multi-dimensional loop indices, which may indicate the current slice, spoke, or frame.

### 2.2 Online Sequence

For online acquisition, we compile BART as a dynamic library and link it with a vendor-specific driver sequence that can interpret the generated sequence events. A flow chart of the interaction between the library and driver is shown in Figure 2.

The interface is designed in a generalized way to completely disentangle the actual sequence logic from the driver sequence to ensure the flexibility for further development of new sequence types without modifying the driver sequence. The complete interface to the vendor-dependent driver sequence is described by four C headers: *event.h, seq.h, helpers.h, custom ui.h*. To ensure modularity, internal data structures of the sequence are not exposed to the driver sequence. Hence, sequence libraries implementing new sequences can be linked to the driver sequence without any need to update this vendor-specific component. Compatibility of driver sequence and library is ensured by a mutual version check at run-time (bart_seq_version_check) which is performed during an initial handshake. To avoid mistakes, a specific sequence library must also be paired to the driver sequence using a digital signature.

Vendor-specific user interface (UI) parameters are mapped to sequence parameters using a generalized interface that also allows the definition of custom parameters. For every parameter change, feasibility of the configuration set is checked. During execution, sequence events are computed just-in-time and interpreted/converted to actual vendor-specific hardware instructions for every sequence block. The generated block is typically designed as a modular functionality, e.g. a spoiled GRE imaging consisting of an excitation pulse, gradients for k-space encoding and ADC for acquisition.

An exemplary implementation of an imaging block is shown in Figure 2. Prior to event preparation, the start times are determined. The RF phase used for excitation pulse and ADC and the radial projection angle are computed. Gradients are prepared as trapezoids and a failed preparation returns an error, feasible gradient amplitudes are projected onto corresponding axes. Preparation of RF pulses include mapping to a shape and complex modulation depending on the spoiling phase and the slice shift. Within a block, various feasibility checks can be included. Analogous to the pulse, complex modulation of the ADC phase depends on the spoiling phase and in-plane FOV shift. In order to save the acquired data at the correct position, the current looping positions are added to the ADC event.

For reproducibility of acquisitions, a log file containing a synthesized command-line is automatically saved each time a sequence is started. Also, one can extract all required data from the exported raw data. This information set combined with the actual implementation is used to reproduce an identical sequence, which ensures that all information that may be required for (model-based) reconstruction is faithfully preserved.

### 2.3 Offline Sequence Tool and Pulseq Export

Timings and waveforms of a sequence can also be computed offline using the bart seq command based on a high-level parameter set as shown in 3.3 and 3.4. Within this command, the sequence is interpreted as in the driver sequence. Outputs of the command currently include gradient waveforms and k-space positions, as well as k-space position, time, and phase of each ADC sample. This allows extraction of the k-space trajectory for image reconstruction and retrospective computation of FOV shifts.

The events can also be saved in Pulseq format. Continuous gradients and pulse shapes are discretized on a raster. ADC events include additional metadata information for labeling. Trigger events are described via the corresponding Pulseq extension type. For more complicated sequences with multiple ADCs, we split blocks during conversion to the Pulseq format. Data can then be acquired with vendor-specific Pulseq interpreters. The complete command-line used for sequence generation is also stored in the Pulseq file.

## 3 Example Applications

We demonstrate the framework using examples of increasing complexity. As a basic example, we demonstrate an online sequence for radial MRI with full support for in-plane FOV shifts. Here, the trajectory used for reconstruction is derived from the sequence implementation. To show feasibility of the framework for the development of advanced computational MRI applications, we implemented two quantitative MRI applications that combine radial acquisition with model-based reconstruction. The first quantitative method uses linearized subspace reconstruction for *T*_1_ mapping with Inversion-Recovery FLASH, while the second uses a non-linear model-based approach for joint mapping of $R_{2}^{*}$ and *B*_0_ using multi-echo radial FLASH.

For each example, we validate offline reproducibility by comparing the results from measurements performed online with results from a repeated measurement based on an exported Pulseq file. The differences are compared to test-retest differences of the same acquisition strategy.

### 3.1 Data Acquisition

MRI experiments were performed on a 3 T Magnetom Vida (Siemens Health-ineers, Erlangen, Germany) with Pulseq v1.5.1 and the dynamically linked BART sequence.

Phantom experiments were conducted with a commercial reference phantom (CaliberMRI Inc, Boulder, CO USA) consisting of 14 *T*_1_ spheres. Phantom and brain studies were conducted with a 20-channel head/neck coil and cardiac experiments were performed with a combined thorax and spine coil with 26 channels. Brain and cardiac scans were performed on a healthy volunteer (male, 29 years) with written informed consent.

The implemented sequence utilizes a radial FLASH readout with rational approximation of golden angles [31]. Data was acquired with a base resolution of 256, FOV of 220x220 mm^2^ (phantom/brain) or 256x256 mm^2^ (cardiac), slice thickness 6 mm (3 mm brain), and excitation flip angle of *α* = 6° (20° brain). Measurements were acquired within ≈ 4 *s* resulting in a total number of 1300 excitations for cardiac (*T_R_* = 3.11 *ms*), 1200 for phantom (*T_R_* = 3.4 *ms*), and 133 for multi-echo experiments (*T_R_* = 31 *ms*).

### 3.2 FOV Shift for Non-Cartesian MRI

An in-plane FOV shift can generally be realized for non-Cartesian MRI by applying a phase modulation [32] Θ(t)=2πk(t)⋅Δrdτ to the acquired data with ***k*** the k-space trajectory and Δ***r*** the FOV shift. This modulation can be applied directly during Cartesian and radial data during the acquisition by setting the frequency and phase used for demodulation. For online acquisitions this is done in the driver sequence resulting in intrinsically correct complex demodulation. For offline acquisition of non-Cartesian sequences with Pulseq, the data is affected by phase inconsistencies. This can be corrected retrospectively by adding the actual FOV shifts to the bart seq command and using a script provided as part of the BART toolbox.


bart seq -r 377 … -s 0.0:0.1:0.0 adc # shift of 100mm in y 



$BART_TOOLBOX_PATH/scripts/pulseq_correction.sh -s 4.6E-6 adc corr



bart fmac ksp corr ksp_corr


Pulseq interpreters modulate the ADC frequency and phase as a function of the FOV shift. The calculation is not exactly correct, which results in a delay of ≈ 4.6 *μs* (half of the gradient raster time and additional adjustment) [33]. To correct this, timing information and complex phase computed by BART can be used. The complex phase is resampled for each readout line while taking the delay into account and the k-space data is then multiplied with a complex phase.

In the first experiment, differences of raw k-space data from isocenter placed FOV were compared for online and Pulseq sequences. For each method, two phantom measurements with continuous radial readout of five frames with 377 spokes were performed. Global phase changes are expected because temperature changes can lead to global *B*_0_ drifts. An optimal global complex scaling factor was therefore applied to each subtrahend to remove this effect. In a second experiment, images were reconstructed with NLINV [34] and compared for isocenter and FOV-shifted acquisitions for phantom and in vivo measurements. For Pulseq acquisitions, phase inconsistencies due to the FOV shift were corrected retrospectively by providing the FOV-shift as input to the bart seq command and applying the phase modulation to the data in a preprocessing step.

### 3.3 *T*_1_ Mapping with Inversion-Recovery Radial FLASH

The single-shot sequence for T1 mapping starts with a non-selective adiabatic inversion pulse, followed by a continuous radial FLASH readout with random RF phase spoiling [35–37]. Cardiac measurements were conducted under breath-hold and the inversion pulse was ECG-triggered to the early diastolic phase [38, 39]. The cardiac sequence was generated with the following command:


bart seq --trigger --trigger-delay 0.4 --IR NON --slice thickness 6E-3 \



     --FOV 0.256 --BR 256 --dwell 4E-6 --rf duration 620E-6 --FA 6 \



     --BWTP 3.8 --flash --raga -r377 -t1350 --TE 1.9E-3 --TR 3.11E-3 \



     -R raga_indices adc_samples gradients moments ir_cardiac.seq


We performed subspace reconstruction where the signal evolution is constrained to a low dimensional linear subspace [28, 40–44]. The Look-Locker model [45] is given by

(1) $$M\left(t,x_{p}(r)\right)=M_{ss}(r)-\left(M_{ss}(r)+M_{0}(r)\right)e^{-t/T_{1}^{*}(r)},$$


bart signal -F -I -1 $T1_MIN:$T1_MAX:$N1 -5 $FA_MIN:$FA_MAX:$N2 \



        -r$TR -n$SPOKES --short-TR-LL-approx dicc


with the model parameters $x_{p}(r)={\left(M_{0},M_{ss},T_{1}^{*}\right)}^{T}$ where $M_{ss}/M_{0}$ are the steady-state and equilibrium magnetization and $T_{1}^{*}$ the apparent longitudinal relaxation time. We computed a signal dictionary for variable *T*_1_ and nominal flip angles and performed an PCA to extract the first four left-singular basis vectors (*B_c_(t)*). The signal is approximated by a linear combination with corresponding coefficient maps $x_{c}(r)$:

(2) $$M\left(x_{p}(r),t\right)\approx \overset{N_{c}}{\underset{c}{\sum }}x_{c}(r)B_{c}(t).$$


bart svd dicc - S V | bart extract 1 0 N_*C*_ - basis


We precomputed coil sensitivities (𝒞) with subspace-NLINV [46] and solved the linear problem to estimate the coefficient maps.


bart nlinv -g -i12 -B basis -t traj ksp tmp coils



(3) $$\overset{ˆ}{x_{c}}=arg\;\underset{x_{c}}{min}{\left\|𝒫\;ℱ\;𝒞\;𝒟\;ℬ\;x_{c}-y\right\|}_{2}^{2}+\lambda ℛ\left(x_{c}\right)$$


bart pics -geH -i250 -RW:3:64:3e-3 -B basis -t traj ksp coils coeffs


with the temporal basis ℬ, a temporal mask 𝒟 selecting diastolic data, the Fourier transform ℱ, the sampling pattern 𝒫 and ℛ a regularization operator, e.g. joint $\ell_{1}$-wavelet. The physical parameters were fitted pixel-wisely according to

(4) $${\hat{x}}_{p}=\text{arg}\;\underset{x_{p}}{\text{min}}{\left\|{ℬ}^{H}\;𝒟\;ℬ\;x_{c}\;-\;{ℬ}^{H}\;𝒟\;ℳ\left(x_{p}\right)\right\|}_{2}^{2}.$$


bart mobafit -g -B basis -L --init 1:1:0.8 TI coeffs pars


Finally, the Look-Locker correction $T_{1}=M_{0}/M_{ss}T_{1}^{*}+2\;T_{d}$ with a delay time *T_d_* (time between inversion and first acquired spoke) was applied to obtain the *T*_1_ map [47].


bart looklocker -t0 -D$T_*d*_ pars t1map


### 3.4 Model-based $R_{2}^{*}$ and *B*_0_ Mapping with Multi-Echo Radial FLASH

The sequence starts with preparation excitations to achieve a steady-state magnetization, followed by a four-second continuous radial readout. 15 echoes with blip gradients between each readout were used to obtain a uniform k-space coverage. The sequence was generated with the following command:


bart seq --prep_scans 100 --FOV 0.220 --slice_thickness 3E-3 --flash \



     --BR 256 --dwell 46E-7 --rf_duration 13E-4 --FA 20 --BWTP 3.8 \



     --raga -e15 --tiny 26 -r133 --TE 2.55E-3 --TE_delta 1.9E-3 \



     --TR 31E-3 -R raga_ind adc_samples gradients moments meco.seq


The signal evolution in the steady-state for the *m*th echo can be described by

(5) $$S_{{TE}_{m}}\left(x_{p}\right)=\left[\rho_{W}+\rho_{F}\cdot z_{m}\right]\cdot exp\left({TE}_{m}\cdot i2\pi f_{B_{0}}\right)\cdot exp\left(-{TE}_{m}\cdot R_{2}^{*}\right),$$

where the set of unknowns ***x_p_*** contains *ρ_W_* and *ρ_F_* the signals for water and fat, respectively, *f*_*B*_0__ represents the field inhomogeneity and $R_{2}^{*}$ is the effective transversal relaxation rate. [48, 49] *z_m_* describes the 6-peak fat spectrum. [50] A nonlinear forward operator *F* is constructed to map the unknowns of Equation (5) to the acquired multi-channel data ***y*** at TE*_m_*, i.e.,

(6) $$F:x=\left(x_{p},c\right)\mapsto y=𝒫ℱ\left(c⊙S_{{TE}_{m}}\left(x_{p}\right)\right).$$

Also here, 𝒫 is the non-uniform sampling pattern and ℱ the Fourier transform and 𝒞 the coil sensitivities. The estimation of unknown $x={\left(x_{p},c\right)}^{T}$ of Equation 6 is then formulated as the optimization problem solved

(7) $$\hat{x}={\text{argmin}}_{x\in \;D}\underset{\text{TE}}{\sum }\|𝒫ℱ𝒞\cdot S_{{\text{TE}}_{m}}(x)-{Y_{{\text{TE}}_{m}}\|}_{2}^{2}+R(x).$$


bart moba -i20 -g -D -m1 -R3 -o 1. -k --kfilter-2 -C500 -j7e-5 \



      --other echo=TE … -t traj ksp TI reco sens


*D* is a convex set for non-negativity of $R_{2}^{*}$ and R(⋅) is the regularization term for both, parameter maps and coil sensitivity maps. In particular, we use Sobolev regularization on the coil sensitivity maps [34] and the field inhomogeneity $f_{B_{0}}$ map to enforce smoothness and a joint $\ell_{1}$-Wavelet sparsity constraint [38] on remaining parameters to exploit sparsity and correlations between maps.

## 4 Results

Figure 3 shows the differences between measurements using online acquisition and using Pulseq. After correction with complex global scaling factors, the difference of the k-space differences for both are comparable to test-retest results, confirming offline reproducibility of the scan.

In Figure 4, we compare images acquired with radial FLASH again using both variants, Pulseq and online acquisition. When acquiring data in the isocenter, only noise-like differences can be seen between both methods for the phantom scan. When using the vendor-specific Pulseq interpreter with FOV shift, phase inconsistencies cause artifacts. After retrospective correction, the comparison of FOV-shifted and corrected Pulseq data to online acquisitions with inherently correct in-plane shifts again shows only noise-like differences comparable to the isocenter experiment. In a cardiac short-axis experiment, the same effect can be seen, although larger differences between the two measurements can be observed due to physiological changes between the two measurements, which is expected.

T1 maps of the NIST phantom placed in the isocenter show only small visual differences (Figure 5), for both, Pulseq compared to online acquisitions and test-retest of the same acquisition strategy. Evaluation of the 14 *T*_1_ values (ranging from 42 *ms* to 1.87 *s*) results in a mean difference of 0.01 *ms* (standard deviation 1.88 *ms*) between the acquisitions.

In Figure 6 cardiac T1 maps of a Pulseq and online acquisition in two consecutive series are presented, where phase inconsistencies were successfully corrected for the Pulseq datasets. Small residual visual differences are shown in difference maps scaled by a factor of five. Comparison of the two acquisition strategies show similar variability as test-retest with inter-measurement deviations mainly due to physiological changes. A mean difference of 10 ms (standard deviation 20 ms) between the acquisitions is found across six standardized myocardial ROIs [51].

Model-based reconstructions of a FLASH sequence utilizing 15 echoes are shown in Figure 7. Good agreement can be found in the parameter maps for both, $R_{2}^{*}$ and *B*_0_ maps, between Pulseq and online measurements. Again, similar deviations between acquisition methods are observable as for test-retest experiments.

The comparisons of online and Pulseq acquisitions for both applications show good agreement in the parameter maps. A successful correction for off-center measurements is also demonstrated in the cardiac example. Similar T1 maps are obtained for both strategies and differences can be explained with physiological changes, e.g. different breath-hold positions and a different number of spokes in diastolic phase. Good agreement can also be found for the $R_{2}^{*}$ and *B*_0_ maps of the brain multi-echo acquisition where small differences can be observed in the magnified difference maps. For both applications, the differences between Pulseq and online acquisitions are comparable to test-retest differences of the same acquisition strategy.

## 5 Discussion

In this work, we extended BART, a widely used open-source toolbox for image reconstruction, to a comprehensive end-to-end framework for computational MRI that now also includes a sequence development framework. Furthermore, we integrated BART as a library to a vendor-specific interpreter sequence, which enables online parameter adjustment. This driver is currently available for scanners from Siemens Healthineers (Erlangen, Germany). In addition, we implemented an offline tool that can recreate the same sequence and export it as a Pulseq file, suggesting compatibility with Pulseq drivers for various MRI scanners and with simulation frameworks. Newer versions of Pulseq interpreters also address the phase inconsistency problem by including an implementation of the same FOV positioning algorithm directly linked to the scanner’s UI [33].

An integrated framework for sequence development and reconstruction offers many advantages as k-space trajectories, gradient and pulse waveforms, and timing information can be recovered directly from the sequence and used in model-based reconstruction algorithms. As examples for integrated computational MRI method where sequence and model-based reconstruction are tightly integrated, we show examples of highly-accelerated (4 seconds) *T*_1_ mapping and joint $R_{2}^{*}$/*B*_0_ mapping. After online acquisition, an identical sequence can be recomputed, which ensures that all information that may be required for model-based reconstruction is faithfully preserved.

The integration into the development workflow of the BART toolbox ensures long-term reproducibility and maintenance for sequences. Using a continuous integration pipeline, we automatically run tests when the software is changed to be able to identify and fix bugs early on. Tests are implemented on different levels, ranging from unit tests for low-level functionality such as event preparation and block generation to integration tests for the bart seq tool.

In this work, we focus on online acquisition and reproduction of the same sequence for advanced quantitative MRI, which is shown for two applications. As even more advanced applications, a radial arterial spin labeling sequence and chemical exchange saturation transfer technique with joint T1 mapping using dynamic transitions were recently presented [52–54], demonstrating the potential of this framework. A next step is to directly integrate the framework with a Bloch-model based reconstruction [55].

Although not further addressed in this work, online image reconstruction can be implemented using the BART streaming framework as described recently [56] and an example for non-Cartesian MRI is distributed together with our vendor-specific driver sequence. In order to enable online execution also on scanners from other vendors, a corresponding driver sequence needs to be implemented.

## 6 Conclusion

An end-to-end open-source framework for sequence design and model-based reconstruction based BART can be used to develop advanced qMRI techniques. Online integration with a vendor-specific interpreter enables online adjustment of sequence parameters, while Pulseq export allows an exact offline reproduction of the sequence.

## Figures and Tables

**Figure 1:**
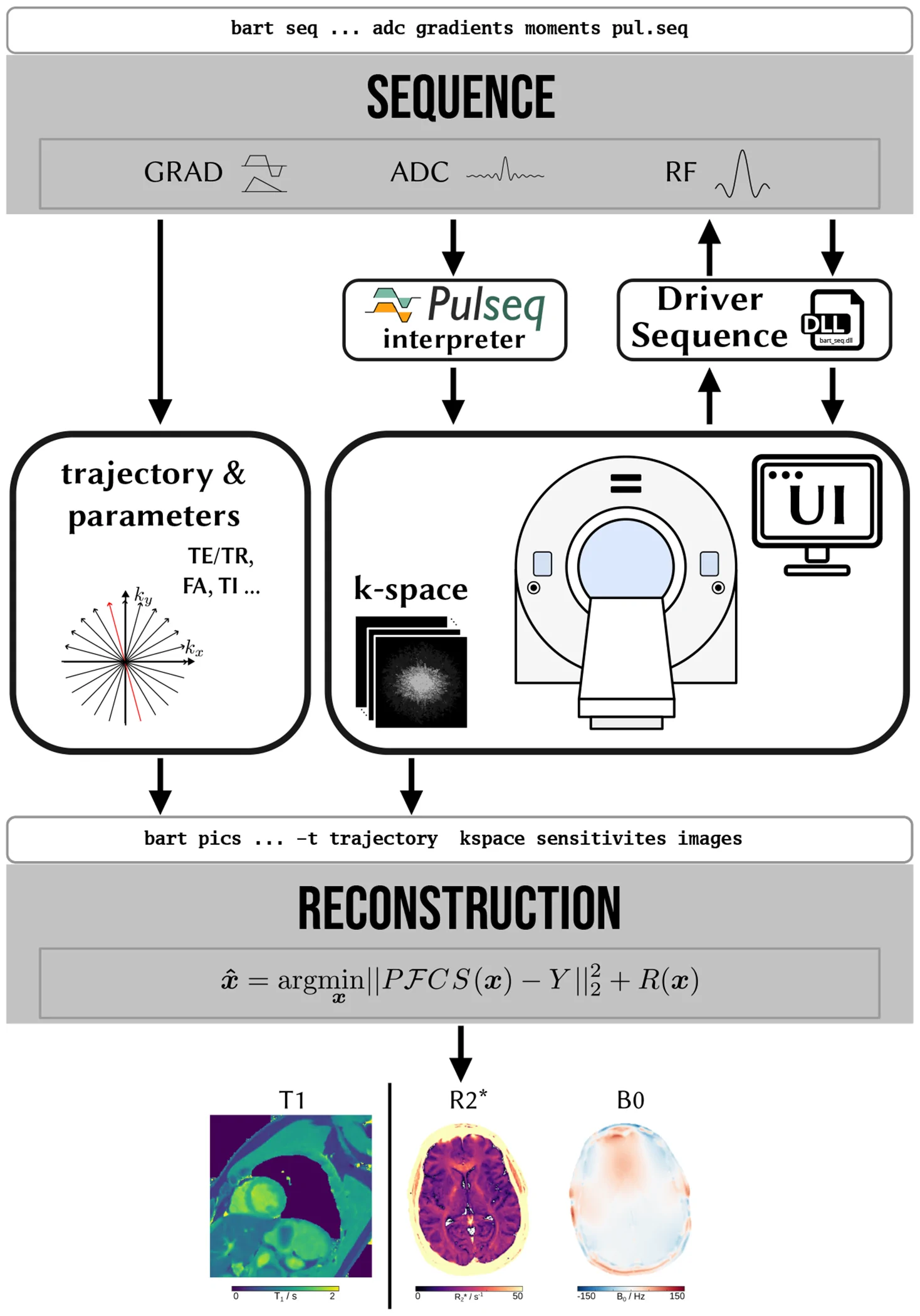
Overview of the proposed open-source computational MRI framework. It is implemented in BART with both, online integration with a driver sequence and offline generation of Pulseq files with the BART seq command. Sequences are uniquely described as a set of events, which are interpreted during the acquisition. Parameters required for image reconstruction, e.g. the trajectory, can be derived from the events. Integration of acquisition and reconstruction into a consistent framework helps to ensure consistency-between both in model-based qMRI.

**Figure 2:**
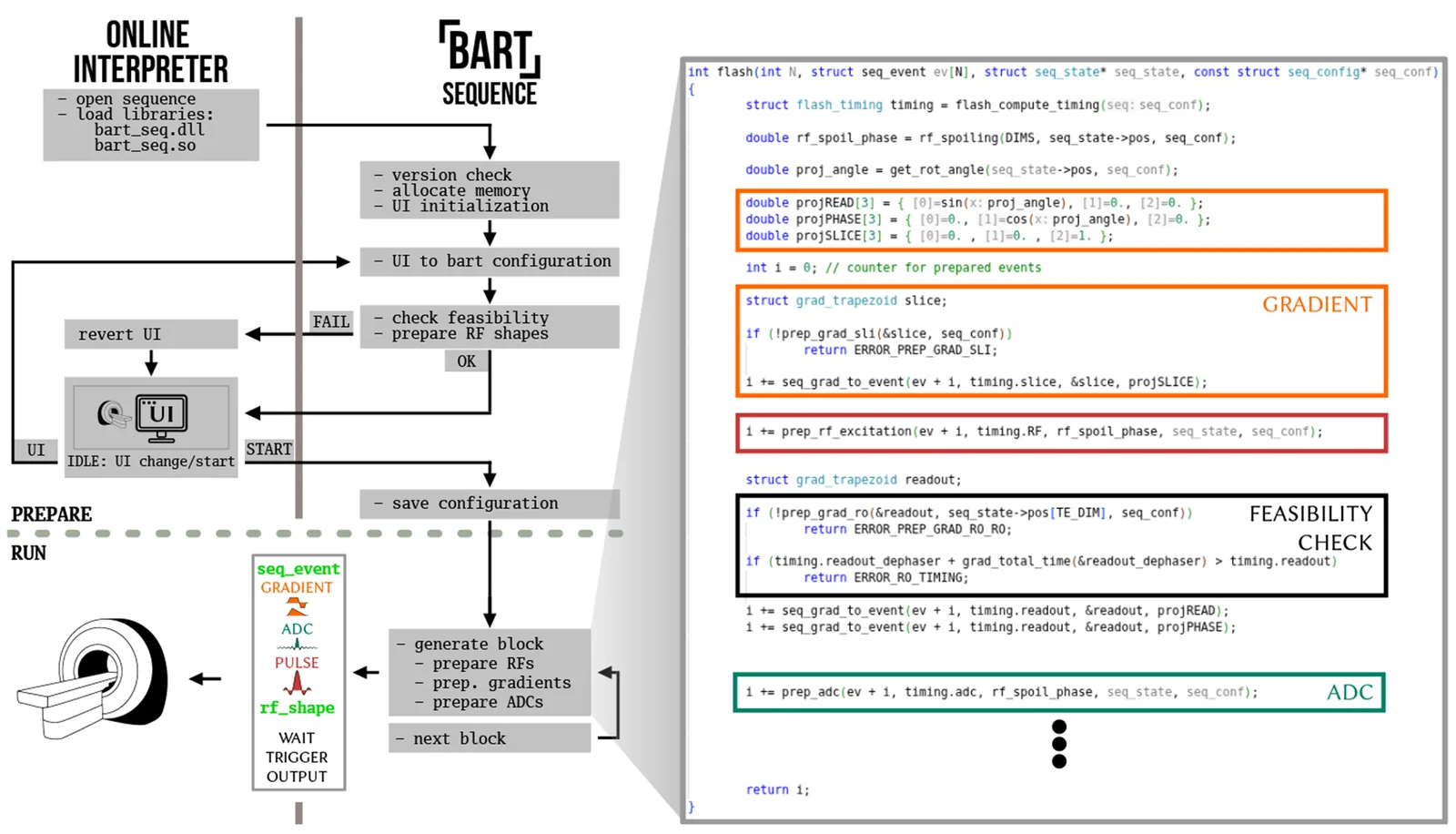
(A) Sequence preparation workflow: opening a sequence starts loading of specific BART library, followed by a version check, memory allocation and initialization of the UI. UI parameters are converted to the sequence configuration and reverted if the sequence is invalid. RF pulses are discretized and prepared in the vendor-specific format. When starting the acquisition, information of the configuration is written to a log file. When running the sequence, events are prepared and interpreted block-wise. (B) Implementation of a gradient-echo imaging block including start times computation, spoiling phase and radial projection angle calculation. Event preparation of trapezoidal gradients with projection to logical axes as well as preparation of RF pulses and acquisition events.

**Figure 3:**
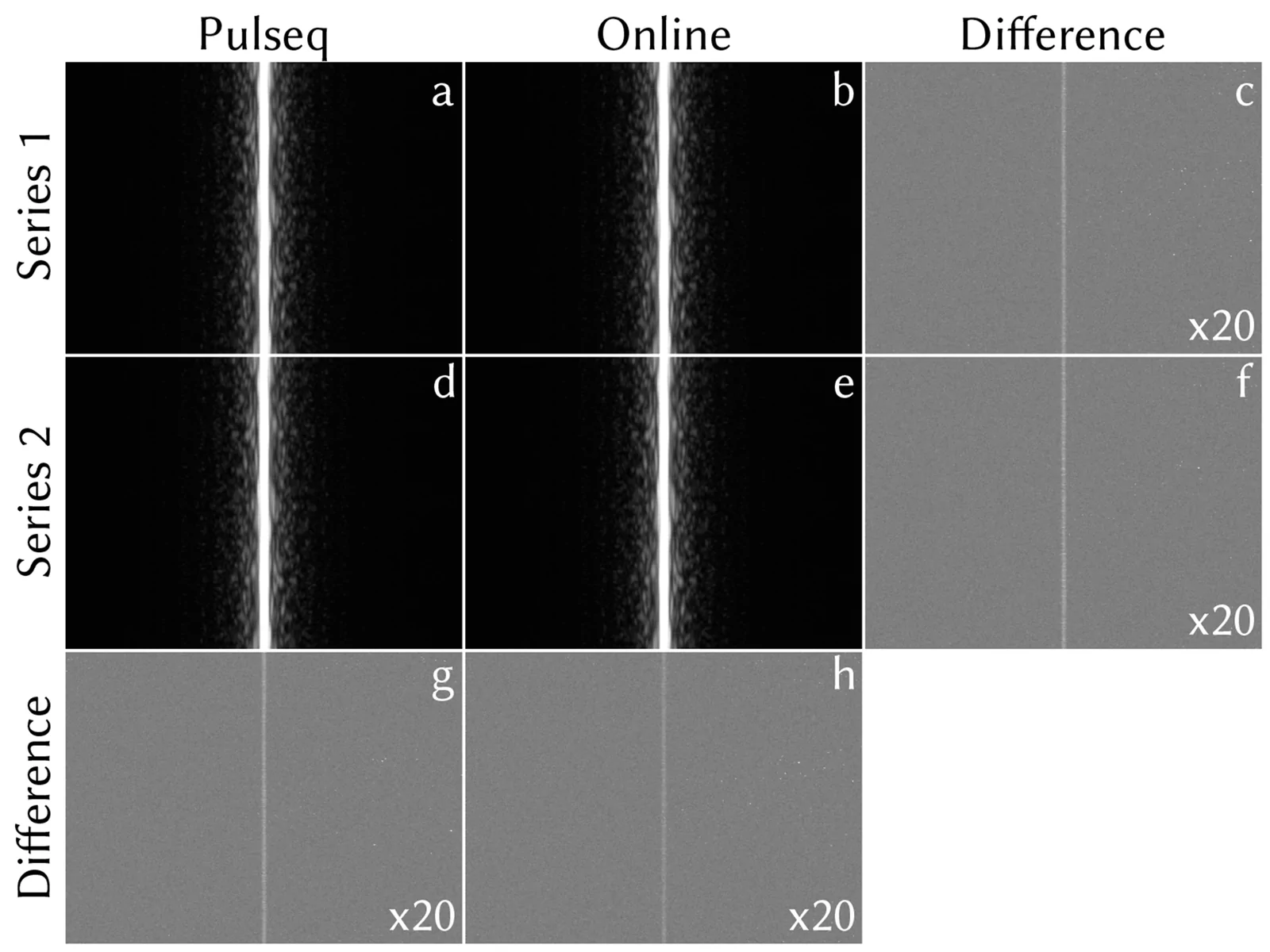
Test-retest experiments show only noise-like differences for both, Pulseq and online acquisitions (g, h). Similarly, small differences can be observed between Pulseq and online acquisition (c, f) with identical sequence parameters.

**Figure 4:**
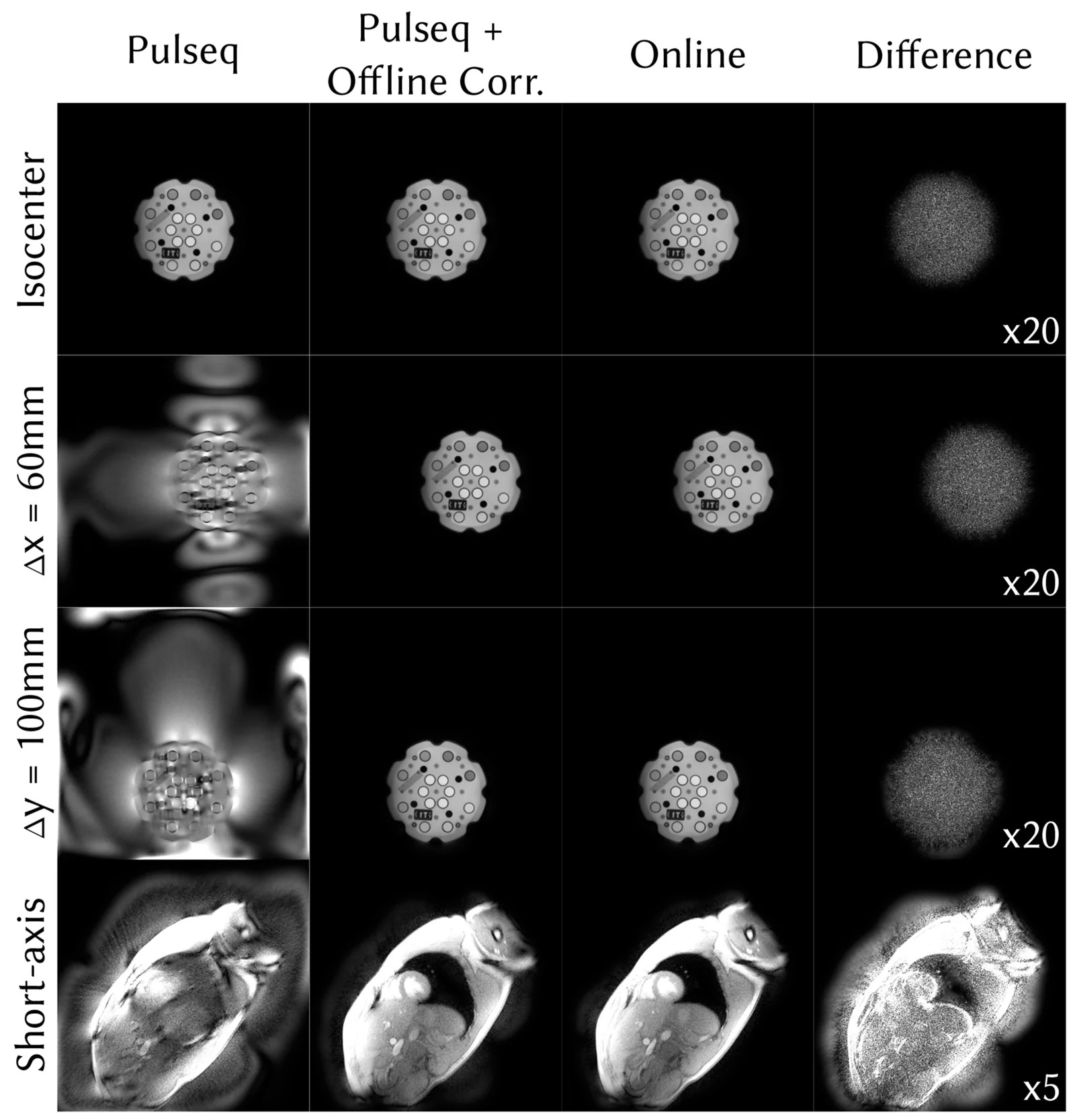
Online and offline Non-Cartesian acquisition with FOV shift. Pulseq acquisitions are affected by phase errors. After retrospective correction, only noise-like differences remain between online acquisition and Pulseq. This is also confirmed for the cardiac measurement, although larger differences remain due to motion.

**Figure 5:**
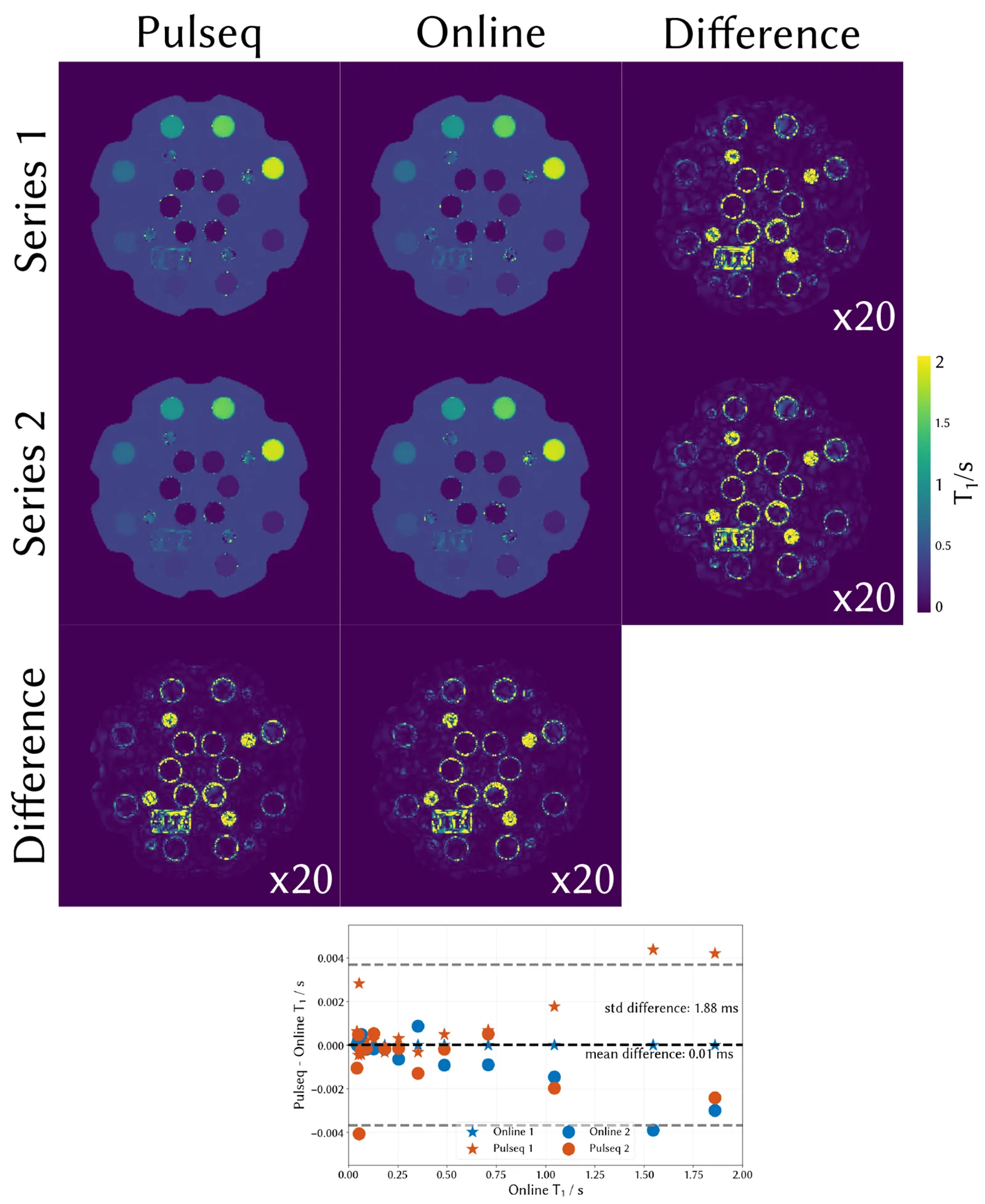
Comparison T1 mapping with a single-shot IR-FLASH sequence with continuous radial readout and subspace-based linear reconstruction of the NIST phantom acquired with Pulseq and online acquisition in the isocenter. Excellent agreement can be found across ROIs within a wide range of T1 values. Again, differences between Pulseq and online acquisitions are similar as test-retest differences of the same acquisition strategy.

**Figure 6:**
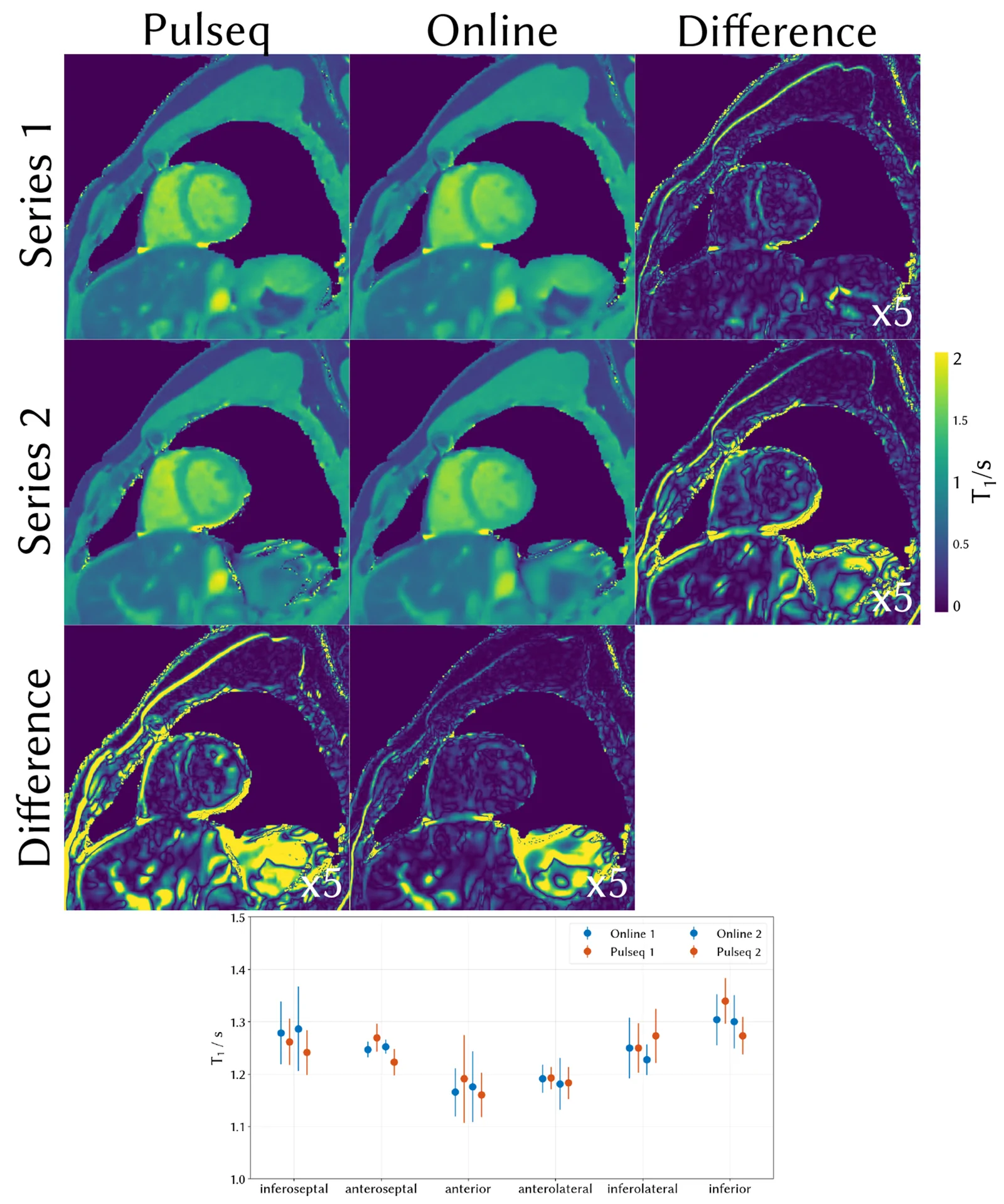
Cardiac T1 mapping with ECG-triggered radial IR-FLASH acquisition and linear subspace reconstruction utilizing Pulseq and online integration. After successful phase correction for the FOV-shifted Pulseq acquisition, good agreement of T1 values can be achieved. Visually, this agreement is confirmed by similar difference maps for test-retest experiments with differences in both, comparison of Pulseq and online acquisitions and test-retest, mainly due to physiological motion.

**Figure 7:**
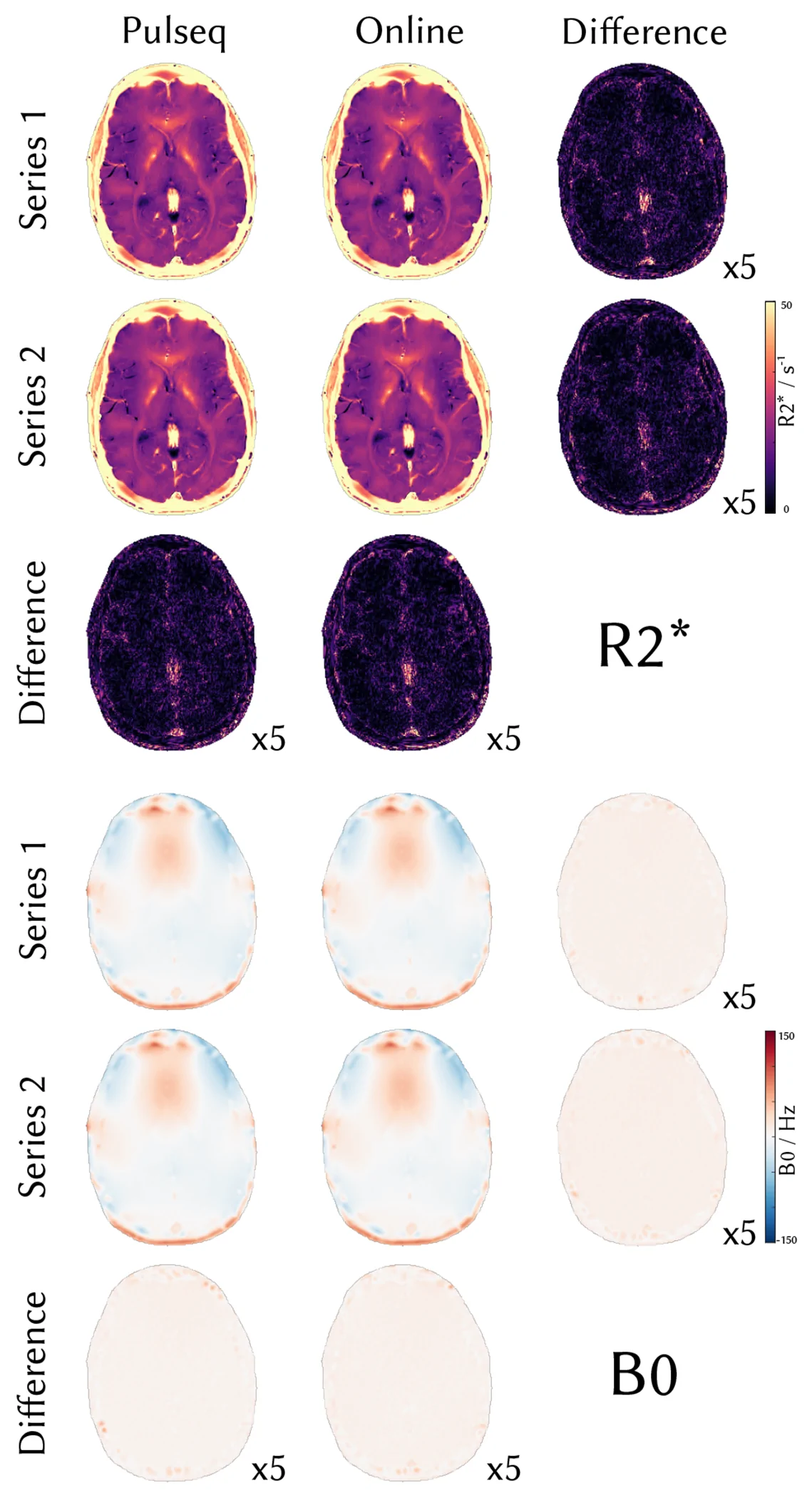
Radial multi-echo data acquisition (4 seconds) and model-based reconstruction for joint $R_{2}^{⋆}$ and *B*_0_ mapping. Good agreement between Pulseq and online acquisitions can be observed for both quantitative maps demonstrated by low differences across the whole brain. This is confirmed by similar differences between test-retest experiments of the same acquisition strategy.

## Data Availability

In the spirit of reproducible research, the code to reproduce the results of this paper is available at https://gitlab.tugraz.at/ibi/mrirecon/papers/bart-sequence. All reconstructions have been performed with BART [57]. BART is available at https://codeberg.org/mrirecon/bart/. The data used in this study is available at Zenodo 10.5281/zenodo.21456517. The Pulseq interpreter can be obtained from the University of Freiburg. The BART Driver Sequence (BOOST: BART Online Open-Source Sequence Toolbox) can be obtained from the Graz University of Technology via Siemens Healthineers’ C2P platform. BART is continuously being developed and maintained since 2013. Functionality of the toolbox and reproducibility of publications is ensured by regular automated testing and a public mailing list is available for support. The Institute of Biomedical Imaging at Graz University of Technology and BART developers are committed to supporting the toolbox in the future.
